# Developmental fates of shark head cavities reveal mesodermal contributions to tendon progenitor cells in extraocular muscles

**DOI:** 10.1186/s40851-021-00170-2

**Published:** 2021-02-15

**Authors:** Shunya Kuroda, Noritaka Adachi, Rie Kusakabe, Shigeru Kuratani

**Affiliations:** 1 Laboratory for Evolutionary Morphology, RIKEN Center for Biosystems Dynamics Research (BDR), 2-2-3 Minatojima-minami, Chuo-ku, Kobe 650-0047, Japan; 2 Department of Biology, Graduate School of Science, Kobe University, Kobe, 657-8501 Japan; 3 Aix-Marseille Université, CNRS, IBDM UMR 7288, 13288 Marseille, France; 4 Laboratory for Evolutionary Morphology, RIKEN Cluster for Pioneering Research (CPR), 2-2-3 Minatojima-minami, Chuo-ku, Kobe 650-0047, Japan

**Keywords:** Head muscles, Head cavity, Head mesoderm, Extraocular muscles, Tendon

## Abstract

Vertebrate extraocular muscles (EOMs) function in eye movements. The EOMs of modern jawed vertebrates consist primarily of four recti and two oblique muscles innervated by three cranial nerves. The developmental mechanisms underlying the establishment of this complex and the evolutionarily conserved pattern of EOMs are unknown. Chondrichthyan early embryos develop three pairs of overt epithelial coeloms called head cavities (HCs) in the head mesoderm, and each HC is believed to differentiate into a discrete subset of EOMs. However, no direct evidence of these cell fates has been provided due to the technical difficulty of lineage tracing experiments in chondrichthyans. Here, we set up an *in ovo* manipulation system for embryos of the cloudy catshark *Scyliorhinus torazame* and labeled the epithelial cells of each HC with lipophilic fluorescent dyes. This experimental system allowed us to trace the cell lineage of EOMs with the highest degree of detail and reproducibility to date. We confirmed that the HCs are indeed primordia of EOMs but showed that the morphological pattern of shark EOMs is not solely dependent on the early pattern of the head mesoderm, which transiently appears as tripartite HCs along the simple anteroposterior axis. Moreover, we found that one of the HCs gives rise to tendon progenitor cells of the EOMs, which is an exceptional condition in our previous understanding of head muscles; the tendons associated with head muscles have generally been supposed to be derived from cranial neural crest (CNC) cells, another source of vertebrate head mesenchyme. Based on interspecies comparisons, the developmental environment is suggested to be significantly different between the two ends of the rectus muscles, and this difference is suggested to be evolutionarily conserved in jawed vertebrates. We propose that the mesenchymal interface (head mesoderm vs CNC) in the environment of developing EOM is required to determine the processes of the proximodistal axis of rectus components of EOMs.

**Supplementary Information:**

The online version contains supplementary material available at 10.1186/s40851-021-00170-2.

## Background

**Fig. 1:**
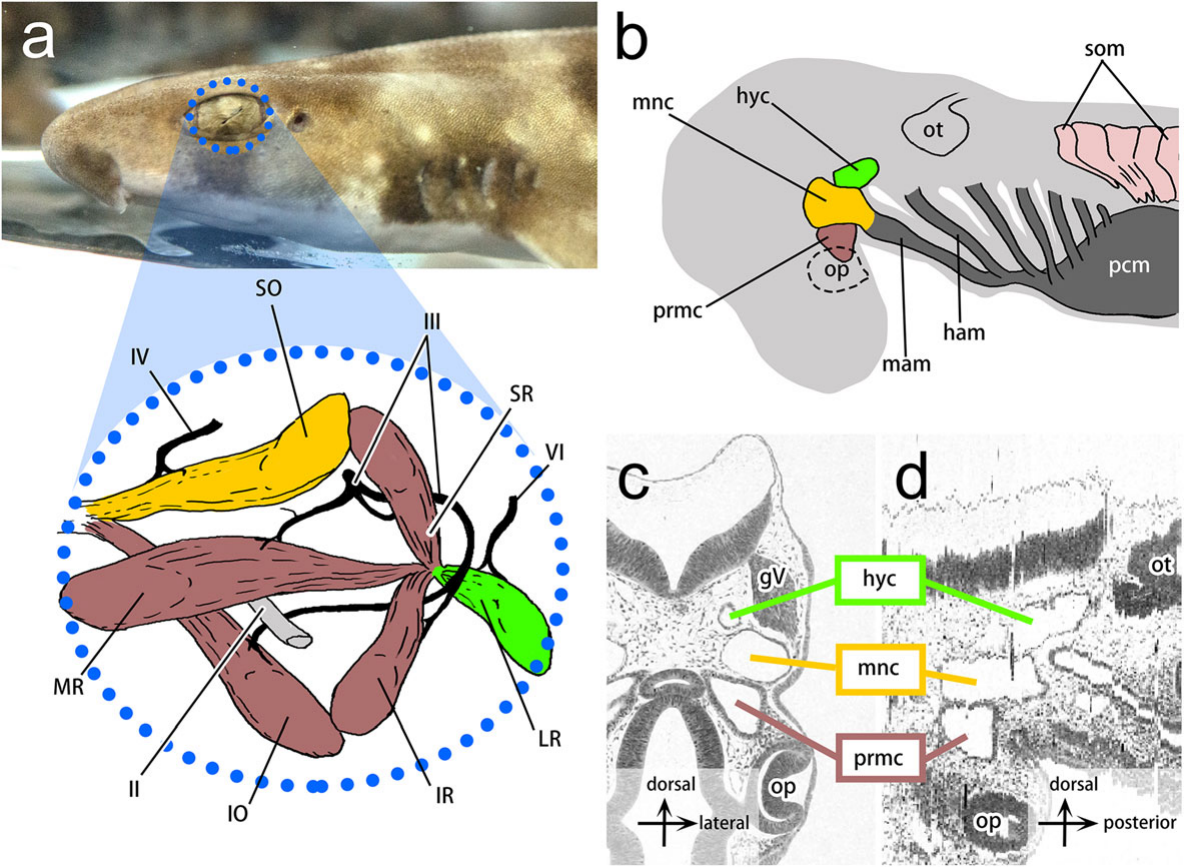
Anatomy of EOMs and distributions of head cavities in the shark embryo. **a** Left lateral view of an adult shark (*S. torazame*; top) and schematic drawing of the left extraocular muscles (bottom). Sets of EOMs innervated by the same cranial motor nerve are shown in the same color: EOMs innervated by the oculomotor nerve in brown, by the trochlear nerve in orange, and by the abducens nerve in green. The blue dotted line indicates the outline of the left eye. **b** A schematic drawing of the left lateral view of the shark embryo at st. 25 showing the positional relationships of head cavities (HCs) to the pharyngeal and cardiac mesoderm (dark gray) and somites (light pink). Each HC is marked in the same color as sets of EOMs in (**a**), which are predicted to be derived from a single HC. **c**, **d** H&E sections in the transverse section (**c**) and the sagittal section reconstructed from serial transverse sections (**d**), showing the anatomical position of HCs, which are arranged in series dorsoventrally (**c**) and anteroposteriorly (**d**). The scheme in (**a**) is modified from ref. [1]. II, optic nerve; III, oculomotor nerve; IV, trochlear nerve; VI, abducens nerve; gV, trigeminal ganglion; ham, hyoid arch mesoderm; IO, inferior oblique muscle; IR, inferior rectus muscle; LR, lateral rectus muscle; mam, mandibular arch mesoderm; mnc, mandibular head cavity; MR, medial rectus muscle; op, optic cup; ot, otic vesicle; pcm, pericardial mesoderm; prmc, premandibular head cavity; SO, superior oblique muscle; som, somite; SR, superior rectus muscle

The extraocular muscles (EOMs) connect the surface of the eye and cranial wall and function in eyeball movements. They consist primarily of four recti and two oblique muscles and are innervated by three cranial motor nerves: the oculomotor, trochlear, and abducens nerves (Fig. 1a). The primordia of EOMs in amniotes emerge from the unsegmented head paraxial mesoderm, in contrast to segmented trunk somites [2, 3]. Moreover, nonmuscular tissues surrounding the primordia of EOMs in the so-called orbital region consist mainly of mesenchymal cells derived from cranial neural crest (CNC) cells [4]. The CNC cells in this region do not have segmental identity, as is seen in the pharyngeal arches, since they are located anterior to the first pharyngeal arch [5–7].

The mesenchymal state of the head mesoderm is commonly seen in most experimental animals throughout development [8, 9]. In contrast, embryos of chondrichthyans (cartilaginous fishes) generally form three pairs of epithelial coeloms called head cavities (HCs) (Fig. 1b-d) [1, 10–13]. The premandibular head cavity (prmc) originates from the prechordal plate, the anteriormost element of the axial mesoderm, that is, from the anteriormost mesoderm in the vertebrate embryo. The mandibular head cavity (mnc) and hyoid head cavity (hyc), on the other hand, are derived from the head paraxial mesoderm (Fig. 1b-d) [12]. Both HCs arise as schizocoels between the neurula and early pharyngula stages [12]. In later stages, histological observations have shown that the epithelial walls of HCs collapse and that the coelomic structures gradually disappear [14]. Each of the HCs was assumed to give rise to a distinct subset of EOMs innervated by a single cranial nerve (Fig. 1a). Therefore, the morphology of HCs has long been believed to serve as a prepattern for EOM morphology [11, 15–17]. However, this predicted lineage of HCs has only been roughly illustrated by histological observations of developmental series of elasmobranch embryos (sharks, skates, and rays) [10, 14]. To elucidate the detailed cell fates of each HC, cell lineage tracing experiments are required. Moreover, the developmental contributions of HCs to nonmuscular tissues also remain to be investigated [11, 12, 18–20].

In the present study, we examined the developmental fates of shark HCs to determine whether each of the HCs gives rise to a different set of EOMs or other musculoskeletal components. The epithelial nature of shark HCs facilitates accurate and highly reproducible labeling of a specific part of the head mesoderm, which in other animals appears only as a cluster of mesenchymal cells without discrete histological boundaries [9, 21, 22]. We performed lineage tracing experiments using a lipophilic fluorescent dye in embryos of the cloudy catshark *Scyliorhinus torazame* (Tanaka, 1908) [23], which has three pairs of HCs [5, 12]. We confirmed that the HCs give rise to EOMs but also discovered that the cell fates of HCs were more complex than classically predicted (Fig. 1). We further revealed that HCs also give rise to tendon progenitor cells for some of the rectus muscles.

## Methods

### Embryo collection

Fertilized eggs of *S. torazame* were collected from tanks of adult sharks kept at 16 °C at RIKEN. Embryos were staged according to a previous study [24]. For section in situ hybridization and immunohistochemistry, embryos were fixed overnight in 4% paraformaldehyde (PFA) (104,005; Merck KGaA, Germany) in phosphate-buffered saline (PBS) (pH 7.4, AM9625; Thermo Fisher Scientific, USA) at 4 °C.

### Molecular cloning and phylogenetic analysis

Total RNA of *S. torazame* was extracted using TRIzol Reagent (15,596,026; Thermo Fisher Scientific), and the corresponding cDNA was synthesized using SuperScript IV Reverse Transcriptase (18,091,050; Thermo Fisher Scientific). PCR was performed to amplify fragments of the *S. torazame Scleraxis* (*StScx*) gene by LA Taq HS (RR042A; TaKaRa Bio Inc., Japan) with specific primers designed based on the prospective *StScx* sequence obtained from Squalomix, the elasmobranch transcriptome database (https://transcriptome.riken.jp/squalomix/) [25]. The PCR fragments were cloned into the pGEM-T Easy vector (A1360; Promega, USA) and sequenced. For phylogenetic analysis, amino acid sequences of orthologous genes from other vertebrate and invertebrate species were compiled from GenBank (http://www.ncbi. nlm.nih.gov/) and Ensembl (http://www.ensembl.org/). Multiple alignments of protein sequences were performed with MAFFT [26] as implemented on the web server of the European Bioinformatics Institute (http://www.ebi.ac.uk/Tools/msa/mafft/) and saved in FASTA format. The resulting alignments were trimmed by trimAl version 1.3 as implemented in Phylemon 2.0 [27] and aligned using ClustalW (http://www.clustal.org/) without gaps. Phylogenetic trees were constructed using the maximum-likelihood (ML) method in PhyML v.3.1 (http://www.atgc-montpellier.fr/phyml/) [28] to confirm the orthology of the *StScx* gene (Fig. S1).

### Fate mapping and embryonic culture in sharks

For the injection of shark embryos at stage 25, eggs were removed from the seawater tank and briefly incubated on ice. A small window was opened on the surface of the egg case just above the embryo. Embryos were then anesthetized with 20 μl of a mixed solution of 1% ethyl 3-aminobenzoate methanesulfonate (MS-222) (E10521; Sigma) and 2% sodium carbonate (1:1 volume:volume). CM-DiI (C7001; Thermo Fisher Scientific, USA) was prepared as previously described [29] and microinjected into HCs by using a microinjector (MN-151; Narishige, Japan). After injection, 200 μl of 0.2% antibiotic-antimycotic mixed stock solution (09366–44; Nacalai Tesque, Inc., Japan) in PBS was added to the surface of the embryo. The eggshell was sealed with a polycarbonate filter (GTBP01300; Merck Millipore, USA) using cyanoacrylate adhesive (Aron Alpha, also known as ‘Krazy Glue’; Toagosei, Japan), to prevent air bubbles from entering the eggshell and to prevent the contents of the egg from protruding through the opening. The injected embryos were left to develop for 6–7 weeks at 16 °C in seawater with 0.2% antibiotic-antimycotic mixed stock solution without aeration. The incubation seawater was replaced multiple times weekly.

### Histological analysis and in situ hybridization

Fixed embryos were dehydrated and embedded in paraffin (Paraplast Plus, P3683; Sigma-Aldrich) at 65 °C. Sections were cut at a thickness of 7 μm. For fluorescence detection of CM-DiI-labeled samples, the deparaffinized sections were washed twice with PBS and incubated for 1 h at room temperature with 4′,6-diamidine-2′-phenylindole dihydrochloride (DAPI) (5 μg/ml, 10,236,276,001; Roche, Switzerland) in PBS. Then, drops of Omnipaque300 (Daiichi-Sankyo, Japan) were added to the sections, and the coverslips were placed. The sections were then imaged by using an Axio Zoom V16 fluorescence microscope (Carl Zeiss, Germany) with an AxioCam MRm digital camera (Carl Zeiss). For immunostaining, the sections were washed with Tris-HCl-buffered saline (pH 7.8, 20 mM Tris-HCl, 150 mM NaCl) containing 1% Triton X-100 (TST), blocked with 5% skim milk in TST (TSTM) for 30 min, and incubated overnight at room temperature with primary antibodies diluted in TSTM. Myosin heavy chain antibody (1/200, A4–1025; DSHB) was used as a primary antibody. After 3 washes in TST (5 min each), the sections were incubated with secondary antibodies in TSTM for 2 h at room temperature. Anti-mouse IgG horseradish peroxidase (HRP) antibody (1/400, F21453; Thermo Fisher Scientific, USA) was used as a secondary antibody. HRP activity was detected using 0.25 mg/ml peroxidase substrate, 3,3′-diaminobenzidine (DAB) (D5905-50TAB; Sigma), in TST with 0.01% hydrogen peroxide. Hematoxylin and eosin (H&E) or Alcian blue staining was performed according to a standard protocol. The sagittal section in Fig. 1d was reconstructed in silico from serial transverse sections using Avizo software version 8.0.1 (Thermo Fisher Scientific). In situ hybridization on paraffin sections for *S. torazame Scx* (GenBank accession number LC430615) was performed as previously described [30]. Counterstaining for in situ hybridization was performed with Nuclear Fast Red (Vector Laboratories, USA). Adjacent sections were analyzed to compare gene expression patterns and distributions of fluorescently labeled cells. Sections in brightfield images were imaged with a BX53 microscope (Olympus, Japan) with a DP74 digital camera (Olympus). Fluorescent images were processed by ZEN software (Carl Zeiss), and all images were assembled in Adobe Photoshop (Adobe Systems, USA) as previously described [31].

## Results

### Long-term cell lineage tracing in *S. torazame* embryos

**Fig. 2:**
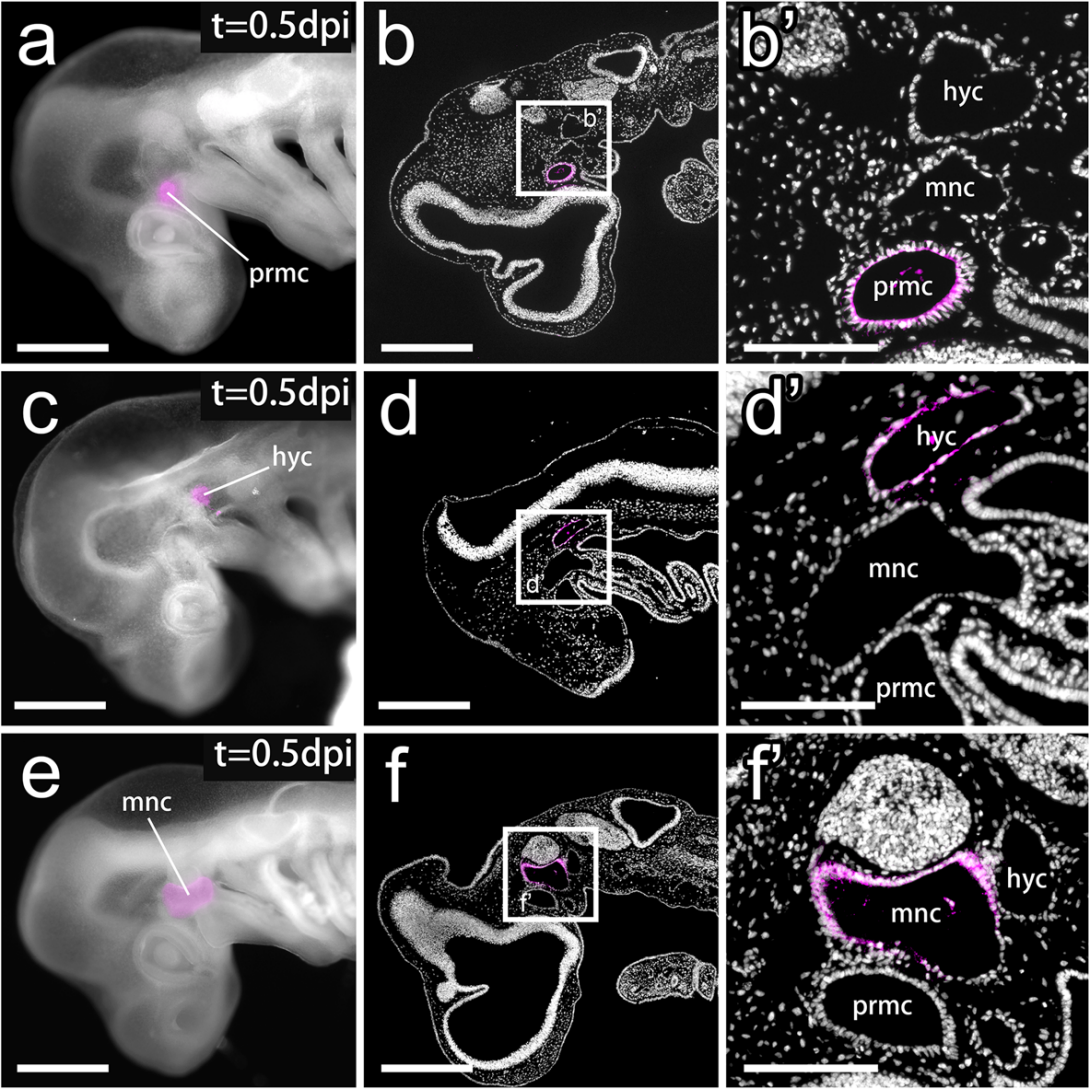
DiI-labeled head cavities at the pharyngula stage. **a-b′** An embryo with DiI injected into the prmc at st. 25. **a** Left lateral view of the DiI-injected embryo at 0.5 days postinjection (dpi). **b-b**′ A sagittal section of (**a**) and a magnified image in the inset (**b**′) showing DiI-labeled cells (magenta) found specifically in the epithelial wall of the prmc. **c-d′** An embryo with DiI injection into the hyc at st. 25. **c** Left lateral view of the DiI-injected embryo at 0.5 dpi. (**d-d**′) A sagittal section of (**c**) and a magnified image in the inset (**d**′) show DiI-labeled cells (magenta) found specifically in the epithelial wall of the hyc. **e-f**′ An embryo with DiI injection into the mnc at st. 25. **e** Left lateral view of a DiI-injected embryo at 0.5 dpi. **f-f**′ A sagittal section of (**e**) and a magnified image in the inset (**f**′) show DiI-labeled cells (magenta) found specifically in the epithelial wall of the mnc. Panels **b**, **b**′, **d**, **d**′, **f**, and **f**′ show sections counterstained with DAPI (gray). hyc, hyoid head cavity; mnc, mandibular head cavity; prmc, premandibular head cavity. Scale bars = 500 μm (**a**, **b**, **c**, **d**, **e**, and **f**), 200 μm (**b**′, **d**′, and **f**′)

In stage 25 (st. 25) *S. torazame* embryos, three pairs of HCs were fully formed but had not yet begun their differentiation into muscle cells (Fig. 1b-d) [12, 14]. We attempted to label the epithelial wall of each HC by microinjecting CM-DiI into the coelom (Fig. 2a, c, and e). Since the left and right sides of the prmc at this stage are connected through a transverse canal just behind Rathke’s pouch [10, 12], when DiI was injected into one side of the prmc, the opposite side of the prmc was also labeled at a certain frequency (7 cases in 12 injected embryos). In contrast, the left and right coeloms of the mnc and hyc were separated from each other (Fig. 1b-d), and we could label each of the HCs specifically with our method. We fixed embryos at 12 hours postinjection (0.5 dpi) and confirmed that CM-DiI labeling was confined to the epithelial wall of the injected coelom (number of times that DiI was specifically recovered in labeled embryos: prmc, *n* = 3/4; hyc, *n* = 5/7; mnc, *n* = 3/5) (Fig. 2). Although the ventral portion of the mnc connected with the tubular pharyngeal arch mesoderm (Fig. 1b), the lumen of the pharyngeal arch canal was almost flattened and very narrowed by this stage [12], preventing ectopic dye labeling outside the mnc. Moreover, no DiI-labeled cells were observed in other tissues around HCs, ensuring exclusive labeling of the HC epithelium (Fig. 2b’, d′, and f′).

**Fig. 3:**
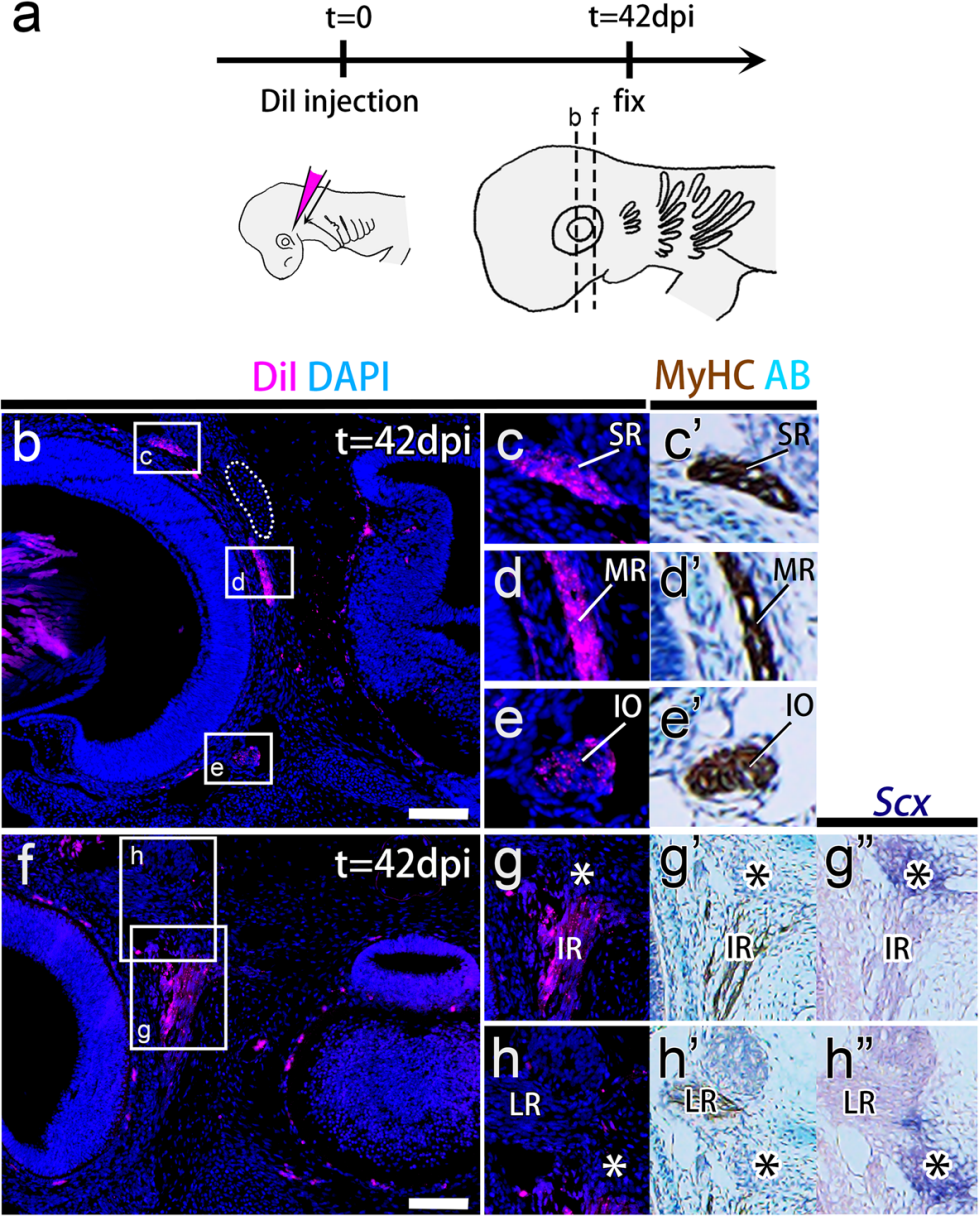
Lineage tracing of the premandibular head cavities. **a** DiI was injected into the coelom of the prmc of a cat shark embryo at st. 25. At st. 31 (42 days postinjection: 42 dpi), the DiI-injected embryos were fixed and histologically examined. **b-h**″ Transverse sections cut through the dashed lines in (**a**) DiI-injected catshark embryos at 42 dpi. Sections (**b**) and (**f**) were each aligned with adjacent sections immunostained with anti-myosin heavy chain (MyHC) antibody (**c**′, **d**′, **e**’, **g**’, and **h**′), stained with Alcian blue (AB) (**c**′, **d**′, **e**’, **g**’, and **h**′), and hybridized in situ with a *Scx* antisense RNA probe (g” and h″). DiI-labeled cells (magenta) are found in muscle fibers of specific sets of extraocular muscles (superior rectus, medial rectus, inferior oblique, and inferior rectus muscles) but not in *Scx*-positive tendon progenitor cells at the attachment site of all rectus eye muscles (asterisks) or in the pila antotica (outlined by the dotted line). IO, inferior oblique muscle; IR, inferior rectus muscle; LR, lateral rectus muscle; MR, medial rectus muscle; SR, superior rectus muscle. Scale bars = 200 μm

In the following experiments, we incubated DiI-injected embryos until st. 31 (42 dpi; Fig. 3a), by which time all EOMs were differentiated and connected to their attachment sites.

We used myosin heavy chain (MyHC) antibody and Alcian blue staining to identify muscles and skeletal tissues in the developed embryos. To visualize tendon progenitor cells, we isolated a shark homologue of the *Scleraxis* (*Scx*) gene, which is known to be expressed in tendon progenitor cells in mice [32], chickens [33], and zebrafish [34]. In situ hybridization analysis confirmed that *S. torazame Scx* expression was specifically detected in cell condensations, which are presumptive tendon progenitor cells, located between the jaw muscle and cartilage (Fig. S2b). Using this *Scx* probe, MyHC antibody and Alcian blue as tissue-specific markers for tendon progenitor cells, differentiated muscles and cartilage, respectively, we examined the distribution of DiI-labeled cells derived from each HC in the established musculoskeletal components, as described below.

### The premandibular and hyoid head cavities give rise to distinct sets of EOMs

**Fig. 4:**
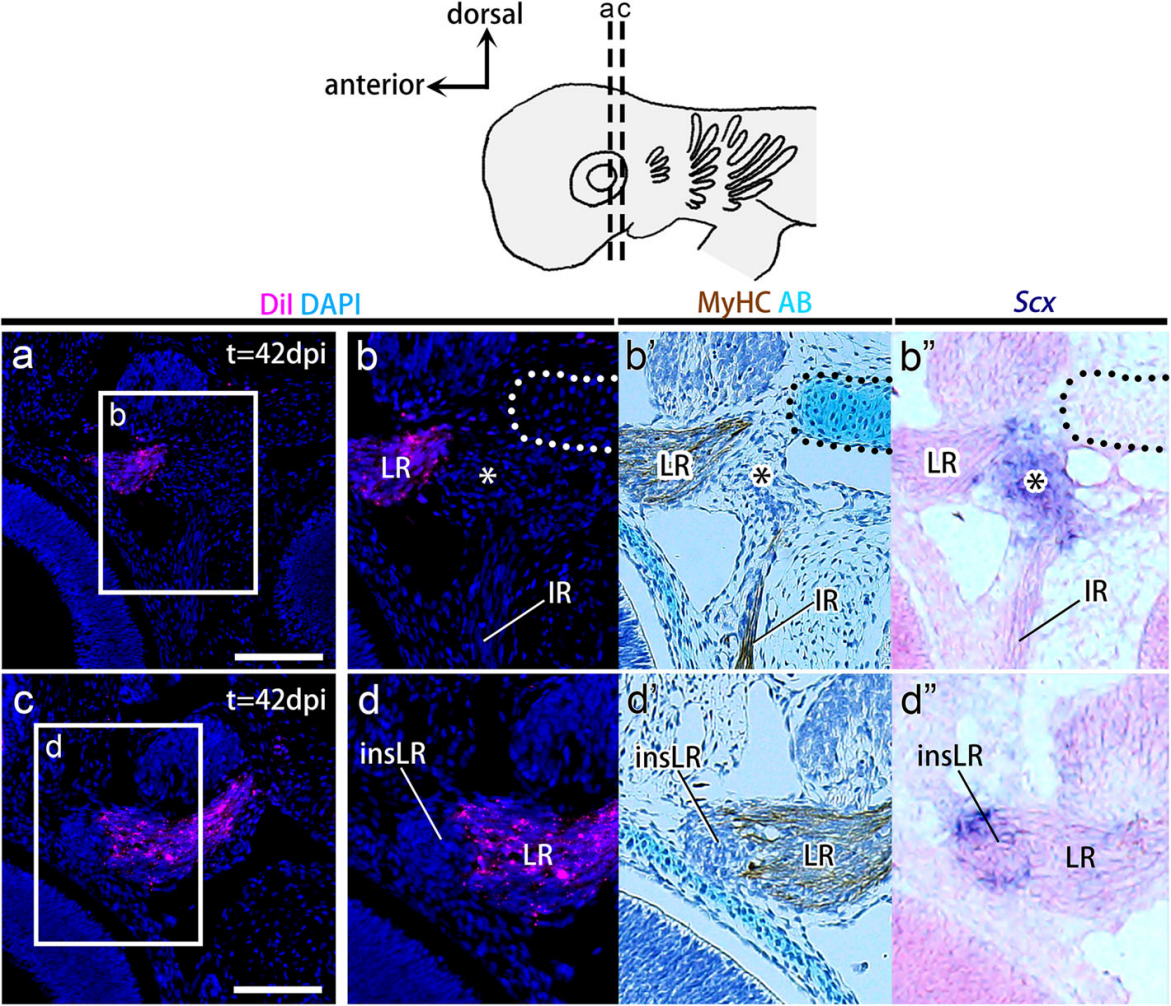
Lineage tracing of the hyoid head cavities. **a-d**″ Transverse sections of CM-DiI-injected embryos aligned with adjacent sections immunostained with myosin heavy chain antibody (**b**′ and **d**′), stained with Alcian blue (AB) (**b**′ and **d**′), and hybridized in situ with a *Scx* antisense RNA probe (**b″** and **d**″). By 42 dpi, CM-DiI-labeled cells (magenta) were found in the lateral rectus muscle but not in *Scx*-positive tendon progenitor cells at the origin of all rectus muscles (asterisks), the insertion site of the lateral rectus muscle (insLR), or the skeleton at the attachment sites of the rectus muscles (pila antotica; outlined by the dotted line). insLR, tendon progenitor cells at the insertion site of the lateral rectus muscle; IR, inferior rectus muscle; LR, lateral rectus muscle. Scale bars = 200 μm

In the embryos injected with DiI into the prmc, labeled cells were observed in muscle fibers of the superior rectus (*n* = 17/19) (Fig. 3c), medial rectus (*n* = 18/19) (Fig. 3d), inferior oblique (*n* = 14/19) (Fig. 3e), and inferior rectus muscles (*n* = 19/19) (Fig. 3g) stained by anti-MyHC antibody (Fig. 3b-h’). DiI-labeled cells were not detected in Rathke’s pouch (*n* = 0/12) (Fig. S3a), the trigeminal ganglia (*n* = 0/19) (Fig. S3b), optic vesicles (*n* = 0/19) (Fig. S3c), sclera (*n* = 0/19) (Fig. S3c), the chondrocranium at the attachment sites of rectus muscles (pila antotica [35, 36];) (*n* = 0/19) (Fig. 3b), or the trabecular cartilage (*n* = 0/19) (Fig. S3d). At the same time, DiI-labeled cells rarely emerged in polar cartilage (*n* = 1/19) (Fig. S3d) and tendon progenitor cells at the origin of the rectus muscles (*n* = 7/19) (Fig. 3g-h”). In the embryos injected with CM-DiI into the hyc, labeled cells were recovered in the lateral rectus muscle fibers (*n* = 15/15) (Fig. 4b, b’, d, and d′). Labeling was undetectable in the trigeminal ganglia (*n* = 0/15) (Fig. S4) and was only rarely detected in the pila antotica (*n* = 1/15) (Figs. 4b-b”, and S4) and two tendon progenitor elements (at the origin of rectus muscles; *n* = 7/15 and at the insertion of lateral rectus muscle; *n* = 8/15) (Fig. 4).

These findings demonstrate that prmc and hyc give rise to the oculomotor nerve- and abducens nerve-innervated components of EOMs, respectively (Fig. 1a), which is in line with previous histological observations (Fig. 1) [17, 37]. Moreover, neither the prmc nor the hyc was suggested to give rise to cartilage or tendon.

### The mandibular head cavity gives rise to tendon progenitor cells as well as muscle fibers

**Fig. 5:**
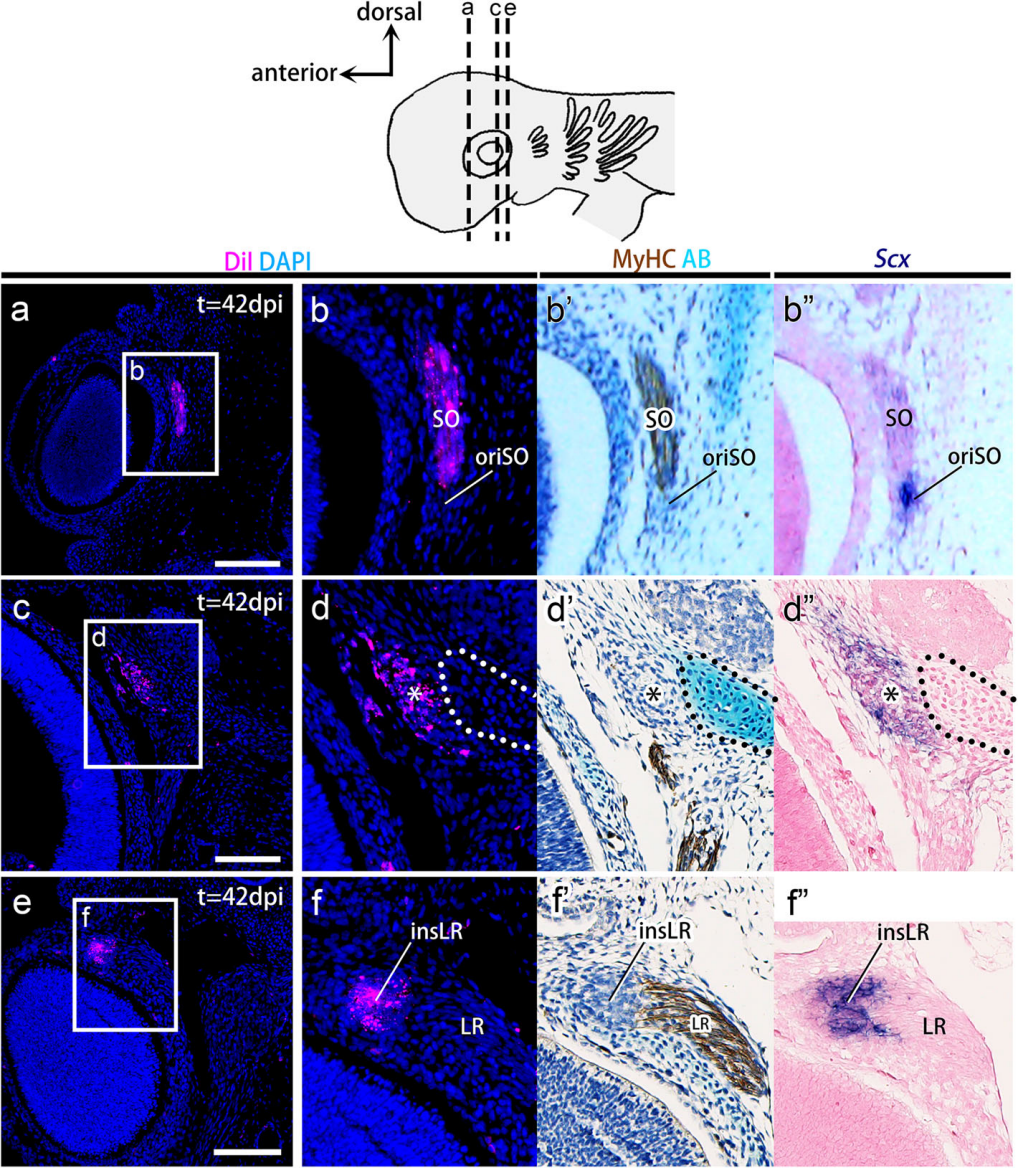
Lineage tracing of the mandibular head cavities. **a-f**″ Transverse sections of CM-DiI-injected embryos aligned with adjacent sections immunostained with myosin heavy chain antibody (**b**′, **d**′, and **f**′), stained with Alcian blue (AB) (**b**′, **d**′, and **f**′), or hybridized in situ with an *Scx* antisense probe (**b**″, **d**″, and **f**″). By 42 dpi, DiI-labeled cells (magenta) were found in the superior oblique muscle, and the *Scx-*positive tendon progenitor cells were found at the scaffold of all rectus eye muscles (asterisks) and the insertion site of the lateral rectus muscle (insLR). insLR, tendon progenitor cells at the insertion site of the lateral rectus muscle; LR, lateral rectus muscle; SO, superior oblique muscle. Scale bars = 200 μm

The labeling of the mnc provided both expected and unexpected results. DiI-labeled cells were recovered in the superior oblique muscle fibers (*n* = 19/22) that were positive for MyHC (Fig. 5a-b’), suggesting that the mnc gives rise to the trochlear nerve-innervated EOM (Fig. 1a). DiI-labeled cells were also detected at the point of origin of all rectus muscles (asterisks in Fig. 5d-d’) and at the insertion site of the lateral rectus muscle (insLR in Fig. 5f-f’) at high frequencies (*n* = 16/22 and *n* = 22/22, respectively). These attachment sites were marked by *Scx* expression (Fig. 5d” and f″), showing the features of tendon progenitor cells (see also Fig. S2). No labeled cells were found in other tendon progenitors (*n* = 0/22) (Fig. 5), the pila antotica (*n* = 0/22) (Fig. 5d-d”), orbital cartilage (*n* = 0/22) (Fig. S5a), trigeminal ganglia (*n* = 0/22) (Fig. S5b), trabecular cartilage (*n* = 0/22) (Fig. S5c), or palatoquadrate cartilage (*n* = 0/22) (Fig. S5d). These results provide the first evidence that the epithelial wall of the mnc in shark embryos, as a part of the head mesoderm, gives rise to specific tendon progenitor cells in addition to trochlear nerve-innervated EOMs (superior oblique muscle; Fig. 1d) [17, 37].

## Discussion

**Fig. 6:**
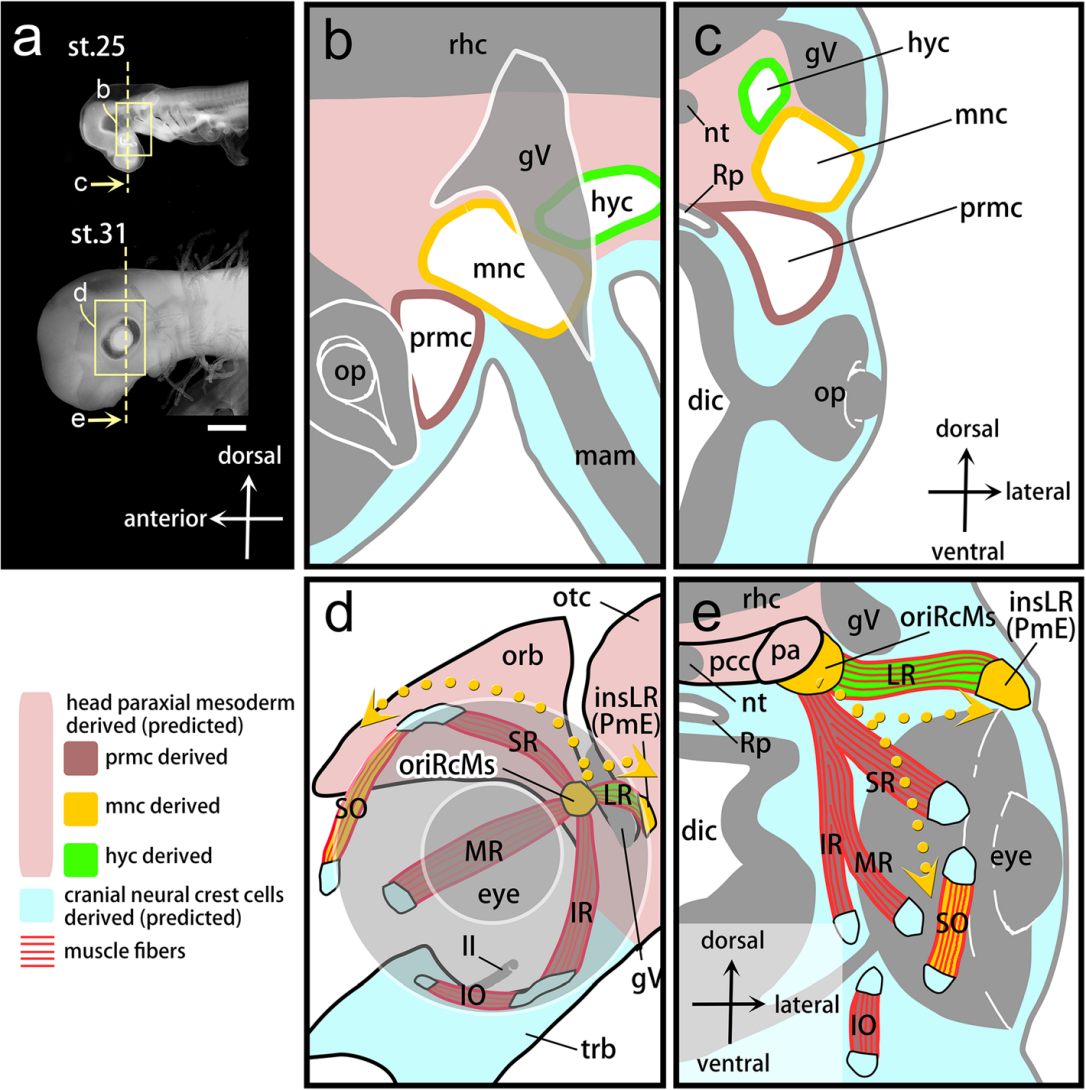
Developmental fates of shark head cavities in the musculoskeletal system of extraocular muscles. **a** Left lateral view of the shark embryos at st. 25 (top) and st. 31 (bottom). **b, c** Schematic drawings of a left lateral view of the inset in (**a**) and a transverse section at the level of the dashed line in (**a**) showing HCs arranged along the anteroposterior axis (**b**) and the dorsoventral axis (**c**) in the embryonic head at st. 25. **d, e** Schematic drawings of a left lateral view of the inset in (**a**) and a transverse section at the dashed line level in (**a**) showing musculoskeletal connections in EOMs via tendons at st.31. The colors of each musculoskeletal component correspond to its developmental origin, shown in (**b** and **c**). Yellow dotted arrows represent the dynamic migratory pathways of mnc-derived cells reconstructed based on the results of the present study. The developmental fate of CNC cell-derived tendons (light blue) and mesodermal chondrocranium (pink) are based on the prediction. II, optic nerve; dic, diencephalon; gV, trigeminal ganglion; hyc, hyoid head cavity; insLR, a tendon at the insertion of the lateral rectus muscle; IO, inferior oblique muscle; IR, inferior rectus muscle; LR, lateral rectus muscle; mam, mandibular arch mesoderm; mnc, mandibular head cavity; MR, medial rectus muscle; nt, notochord; op, optic cup; orb, orbital cartilage; oriRcMs, a tendon at the origin of four-rectus muscles; otc, otic capsule; pa, pila antotica; pcc, parachordal cartilage; pm, PmE, Platt’s ‘muscle E’; prmc, premandibular head cavity; rhc, rhombencephalon; Rp, Rathke’s pouch; SO, superior oblique muscle; SR, superior rectus muscle; trb, trabecular cartilage. Scale bars = 1 mm. Not to scale (**b**-**e**).

In this study, we reported lineage tracing analysis of HCs in shark embryos and showed that each HC gives rise to a set of EOMs innervated by a single cranial motor nerve (Figs. 1d and 6). In addition, our results provide the first evidence that HCs, which belong to the head mesoderm, give rise to dense connective tissues of the head muscles in shark; the mnc gives rise to tendon progenitor cells at the origin of the rectus muscles and at the insertion of lateral rectus muscles (Fig. 6d and e). In contrast, we did not observe any contributions from HCs to the cranial cartilage in our experiments.

Concerning the mnc-derived cellular condensation at the insertion site of the lateral rectus muscle, given its position and developmental origin, it could be the same cell population first described by Platt as ‘muscle E’ (Fig. S6, [14, 15, 38, 39]). Platt and later researchers thought that this condensation gives rise to muscle cells of the distal portion of the lateral rectus muscle (reviewed in [15]). However, in our experiments, this condensation was not positive for the MyHC antibody but did express the *Scx* gene, suggesting that these cells are tendon progenitor cells (Figs. 5 and S6b).

Although the classical studies that predicted the developmental fate of HCs (Fig. 1) were partially supported by our experiments, the actual developmental patterns and processes of the mnc-derived cells turned out to be more complex than was predicted (Fig. 6). Classical studies regarded the pattern of HCs as a prepattern of EOM morphology based on the predicted one-to-one correspondence between an HC and the innervation patterns of EOMs [1, 11, 15, 17]. The results of the present study led us to revise the above hypothesis. One portion of mnc-derived cells retain their original positions throughout development and give rise to tendon progenitor cells at the origin of rectus muscles (Fig. 6). In contrast, the remaining mnc-derived cells show two different migratory pathways, one toward the anterior portion and the other toward the posterior portion of the eye (yellow dotted arrows in Fig. 6d and e). First, the superior oblique muscle primordium, separated from the dorsal part of the mnc, passes above the eye and overtakes the cell population derived from the prmc. At this point, the original anteroposterior arrangement of the HCs was altered. In the posterior part of the eye, the mnc-derived cells give rise to tendon progenitor cells at the insertion of the lateral rectus muscle (Platt’s ‘muscle E’), keeping their leading position relative to the direction of movement of the lateral rectus muscle primordium. It is worth noting here that mnc-derived tendons are not recruited in the muscle attachment of the superior oblique muscles. This may be comparable to the relationship between the syndetome and migratory muscle precursors, both of which are derived from a single somite [40, 41]. However, because the mnc-derived tendons give scaffolds at both ends of the hyc-derived lateral rectus muscles (Fig. 6), it is still unreasonable to compare the relationship between the mnc and hyc with that of two adjacent somites. Overall, we conclude that the morphological pattern of HCs is not a prepattern of EOMs.

### Reevaluating the mesoderm/CNC boundary in the mesenchymal environment in the orbital region

The majority of the mesenchymal component in the vertebrate embryonic head is derived from CNC cells [4, 42]. Since CNC cells differentiate into the pharyngeal skeleton, prechordal cranium, and connective tissues of the head muscles, it has been presumed that musculoskeletal connections in the head would be established through interactions between CNC cells and muscle progenitor cells [4, 42–45]. In heterotopic transplantation of the trunk paraxial mesoderm into the head, grafted cells gave rise to head muscles with nearly normal morphology [46, 47]. In *Tbx1* knockout mice, branchiomeric muscle precursors were absent, but the initial patterning of tendon progenitor cells occurred normally [48]. Thus, the morphogenetic information of the CNC cells that give rise to dense connective tissues in the head can override the identity of the muscle precursors exposed in the ectomesenchymal environment (derived from CNC cells) [49–52].

EOM primordia first appear in the head paraxial mesoderm, where they are detectable by *Pitx2* expression, and subsequently migrate rostrally to enter ectomesenchymal environments in the prechordal region [50, 53, 54]. This migration pattern led to the belief that CNC cells would be the only origin of the dense connective tissues of EOMs, as in the case of other head muscles [4, 42]. CNC cells were confirmed to contribute to some part of the connective tissues of EOMs using chick/quail chimeric embryos [51] and transgenic mice [55]. On the other hand, the skeletal component at the proximal attachment sites (origins) of four rectus components of EOMs is known to be mesodermal in mice and chickens [43, 56]. The latter results suggest that the developing rectus muscles may be on the mesenchymal interface between the head paraxial mesoderm and CNC cells. In the present study, we revealed the contributions of the head mesoderm to tendon progenitor cells at the proximal attachment of rectus muscles in sharks. This result is consistent with the above discussion about the position of the mesoderm/CNC interface in the orbital region. At the same time, this suggests that the corresponding EOM attachment sites in mice (ala hypochiasmatica) and chickens (supratrabecular cartilage) also adopt mesodermal tendons similar to those in shark embryos (Fig. S7). Now, we need to reexamine the cell lineages of connective tissues of EOMs in these two animals, for whom the cell lineages of the structures in the head have been studied in greater detail than in any other vertebrate.

In the present study, we could not perform lineage tracing experiments of shark CNC cells due to technical difficulties. Considering the results of previous experiments in model animals that have shown that the periocular mesenchyme generally consists of CNC cells [55, 57, 58], it is reasonable to speculate that the other tendons in shark EOMs that are not derived from HCs are derived from CNC cells (Fig. 6d and e). Thus, the rectus muscles other than the lateral rectus muscles in sharks are suggested to have CNC-derived tendons at one attachment site and mesodermal tendons at the other sites. The fact that some EOMs have tendons other than CNC-derived tendons suggests that the morphogenetic process of EOMs may be partially free from the identity imposed by the ectomesenchymal environment. Although our results did not support the notion that the morphological pattern of the HCs contributed to that of the EOMs, the cell population boundary in the mesenchymal environment in the orbital region might play some role in establishing the proximodistal axis in the rectus muscles.

Contexts similar to the developmental environment suggested in the present study have recently been reported in some neck and shoulder musculatures. These muscles develop in embryonic environments with mesenchymal boundaries between the CNC and lateral plate mesoderm [59] or cardio-pharyngeal mesoderm [60]. The resultant muscles have heterogenic cell populations of connective tissues. Furthermore, considering the recent report of mesodermal contributions to the posterior part of the pharyngeal skeleton in skate [61] and to the tendon progenitor cells for EOMs in shark (this study), we have to reconsider the rather dualistic view that the morphogenetic processes in head and trunk musculature are regulated strictly by CNC-derived and mesodermal mesenchymal environments, respectively. Further clarification of the developmental mechanisms shared by muscles that develop at the mesoderm/CNC boundary will shed new light on the question of what factors determine the evolutionary coupling or decoupling between the mesoderm/CNC boundary and the morphological boundary in craniofacial and neck-shoulder complexes [56, 62–64].

## Conclusions

In our lineage tracing analysis in shark HCs, we confirmed the classical view of the developmental origin of EOMs; each HC gives rise to different subsets of EOMs innervated by each cranial motor nerve. We also found that the mnc gives rise to tendon progenitor cells at the origin of the rectus muscles and the insertion of the lateral rectus muscle. Given these newly revealed cell fates of shark HCs, we conclude that the previous hypothesis that the EOM developmental pattern was prespecified in HCs should be revised. Our results also suggest that the developmental origins of tendon progenitor cells at either end of most rectus muscles in sharks differ from each other. We speculate that the presence of the head mesoderm/CNC boundary in the mesenchymal environment could be required for establishing the proximodistal axis of the rectus components of EOMs.

## Supplementary Information

**Additional file 1.**

## Data Availability

The newly identified cDNA sequence of the *S. torazame Scx* gene has been registered in GenBank under accession number LC430615. Any other relevant data are available from the corresponding author upon reasonable request.
