# Supplementary material for: Developmental fates of shark head cavities reveal mesodermal contributions to tendon progenitor cells in extraocular muscles
Source: Zoological Lett. 2021 Feb 15;7:3. doi: 10.1186/s40851-021-00170-2 (PMC7885385; doi:10.1186/s40851-021-00170-2)
Supplement: Supplementary file 1 — Additional file 1. [file 40851_2021_170_MOESM1_ESM.docx]

**Additional file 1**

**Developmental fates of shark head cavities reveal mesodermal contributions to the tendon progenitor cells for extraocular muscles**

Shunya Kuroda^1,2^*, Noritaka Adachi^3^, Rie Kusakabe^1^, Shigeru Kuratani^1,4^

^1^Laboratory for Evolutionary Morphology, RIKEN Center for Biosystems Dynamics Research (BDR), Kobe, Japan.

^2^ Department of Biology, Graduate School of Science, Kobe University, Kobe 657-8501, Japan.

^3^ Aix-Marseille Université, CNRS, IBDM UMR 7288, Marseille 13288, France.

^4^ Laboratory for Evolutionary Morphology, RIKEN Cluster for Pioneering Research (CPR), Kobe, Japan.

* Correspondence: shunya.kuroda@riken.jp

**
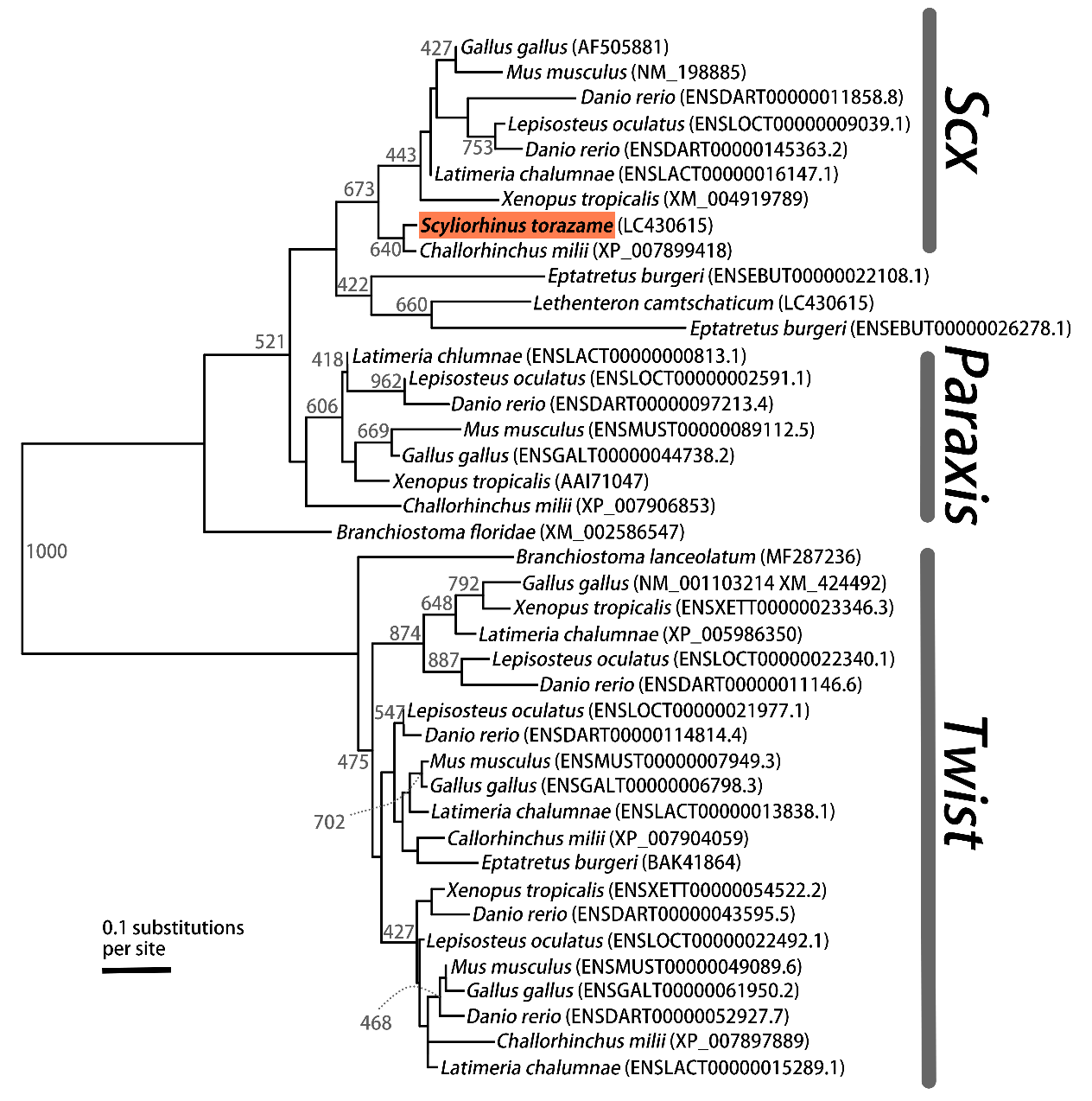
Figure S1. A molecular phylogenetic tree of *Scx, Paraxis,* and *Twist* based on the maximum likelihood method.** Numbers of replicate trees in which the associated genes were clustered together in the bootstrap test (1,000 replicates) are shown next to the nodes if they are greater than 400. The tree is drawn to scale, with branch lengths measured as the number of substitutions per site. Orthology between a newly cloned *S. torazame* *Scx* gene (highlighted in red) and the *Scx* genes of other gnathostomes was supported. Numbers in brackets behind the name of species represent accession numbers.

**
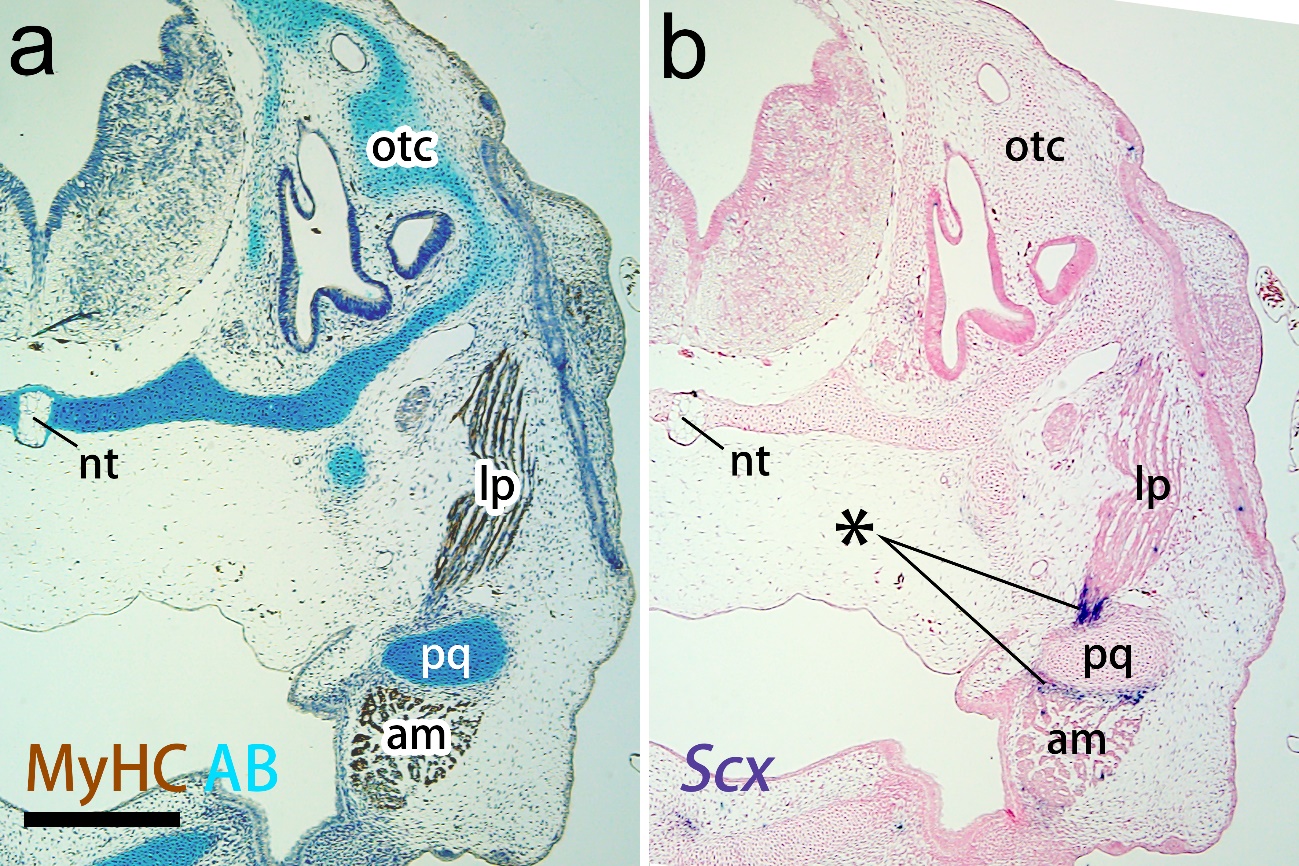
Figure S2. *Scx* expression in the jaw musculature.** Neighboring sections immunostained with MyHC antibody combined with alcian blue (AB) staining (a) and hybridized with *Scx* antisense RNA probe (b) of the st.31 shark embryo. *Scx*-positive cells (asterisk in b) are found specifically in cell condensations at the attachment sites of skeletal muscles to the jaw skeleton.

am, adductor mandibulae muscle; lp, levator palatoquadrate muscle; nt, notochord; otc, otic capsule; pq, palatoquadrate cartilage.

Scale bar = 500 µm.

**
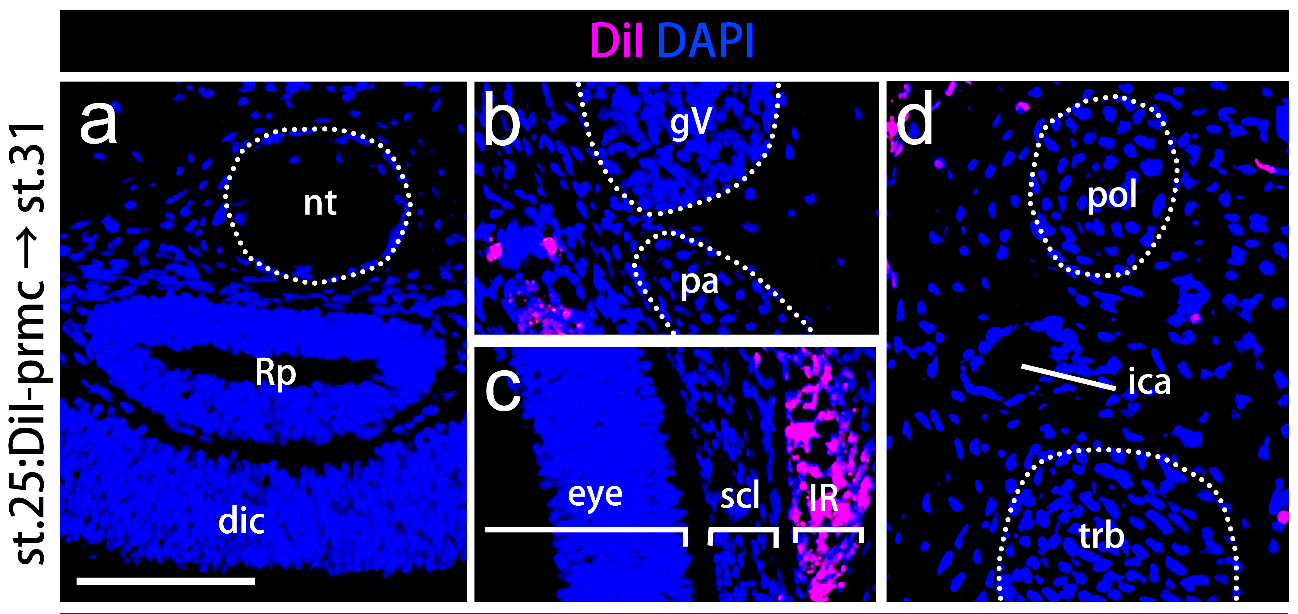
Figure S3. Distribution of DiI-labeled cells in prmc-labeled embryos and their surrounding tissues.** No DiI-labeled cells (magenta) are found in the Rathke’s pouch (a), trigeminal ganglia (b), scleral mesenchyme (c), trabecular cartilage, or polar cartilage (d) of st. 31 embryos.

dic, diencephalon; gV, trigeminal ganglion; ica, intracarotid artery; IR, inferior rectus muscle; nt, notochord; pa, pila antotica; pol, polar cartilage; Rp, Rathke’s pouch; scl, sclera; trb, trabecular cartilage.

Scale bar = 100 µm.

**
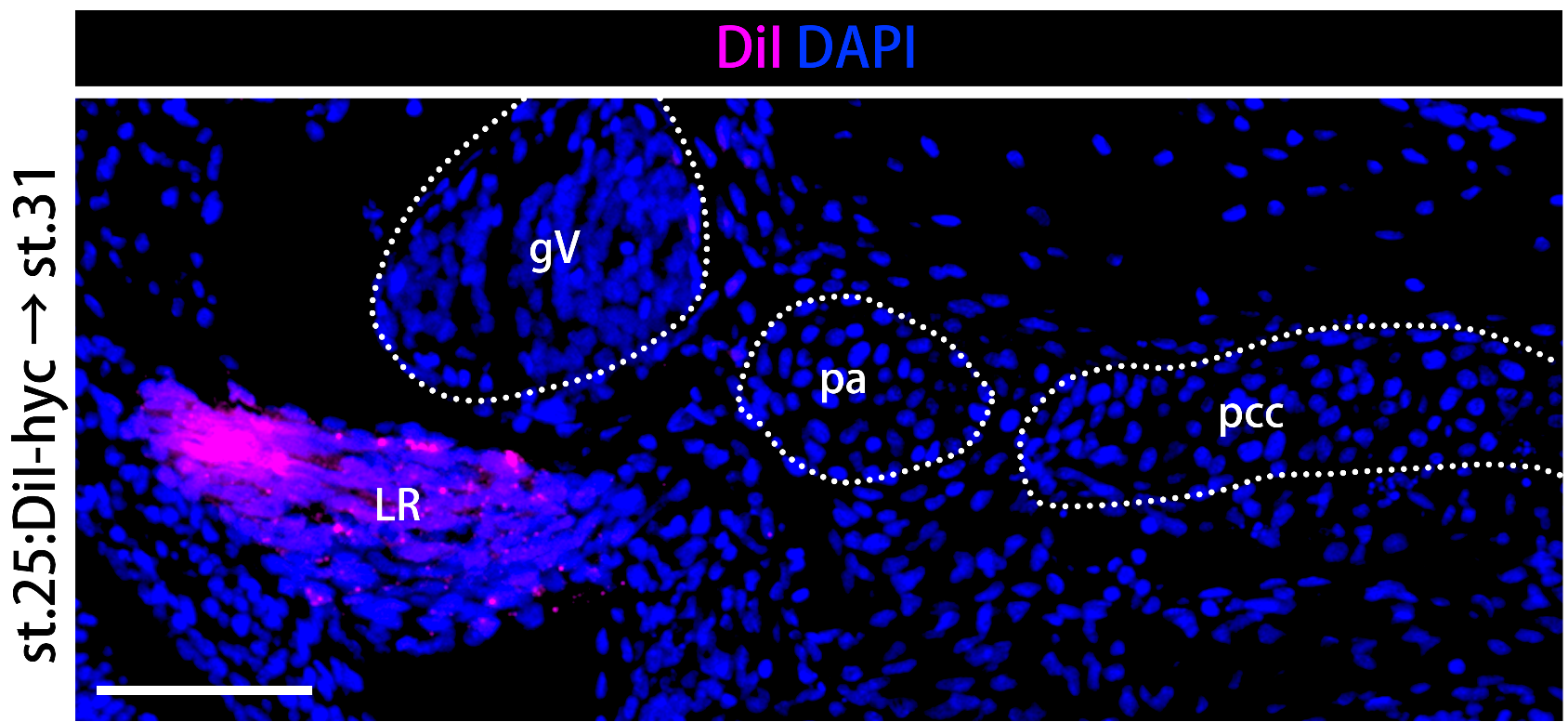
Figure S4. Distribution of DiI-labeled cells in hyc-labeled embryos and their surrounding tissues.** No DiI-labeled cells (magenta) are found in the trigeminal ganglia, pila antotica, or parachordal cartilage.

gV, trigeminal ganglia; LR, lateral rectus muscle; pa, pila antotica; pcc, parachordal cartilage.

Scale bar = 100 µm.

**
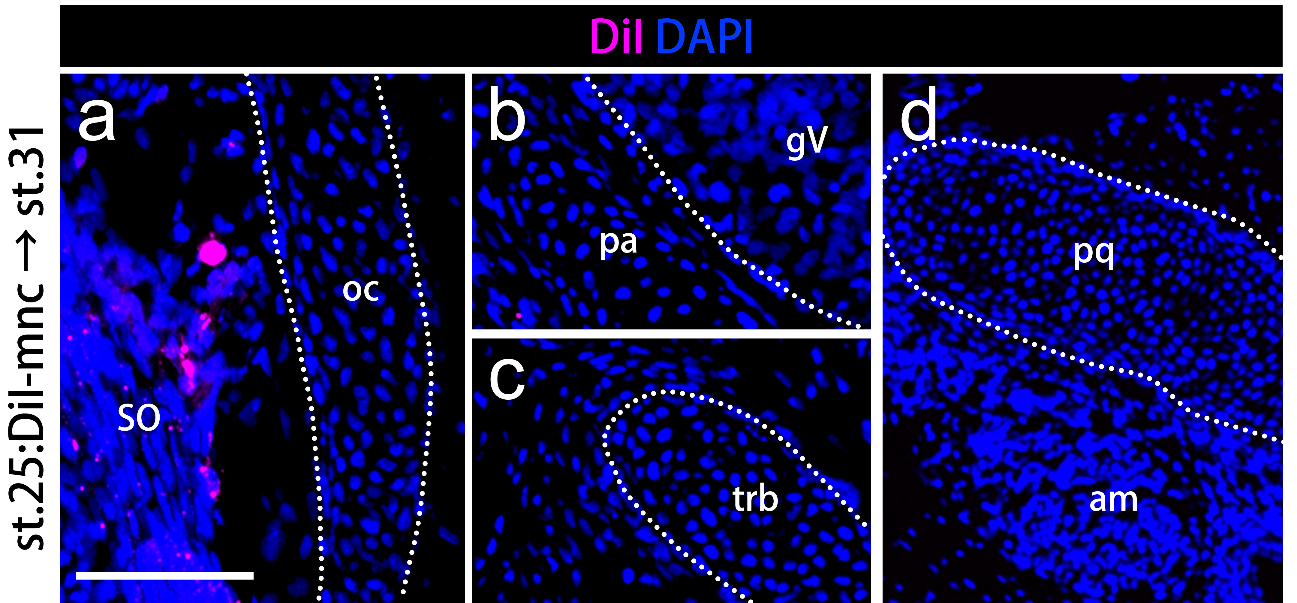
Figure S5. Distribution of DiI-labeled cells in mnc-labeled embryos and their surrounding tissues.** No DiI-labeled cells (magenta) are found in the orbital cartilage (a), pila antotica (b), trigeminal ganglia (b), trabecular cartilage (c), palatoquadrate cartilage (d), or adductor mandibularis muscle (d) at st. 31.

am, adductor mandibularis muscle; gV, trigeminal ganglia; oc, orbital cartilage; pa, pila antotica; pq, palatoquadrate cartilage; SO, superior oblique muscle; trb, trabecular cartilage.

Scale bar = 100 µm.

**
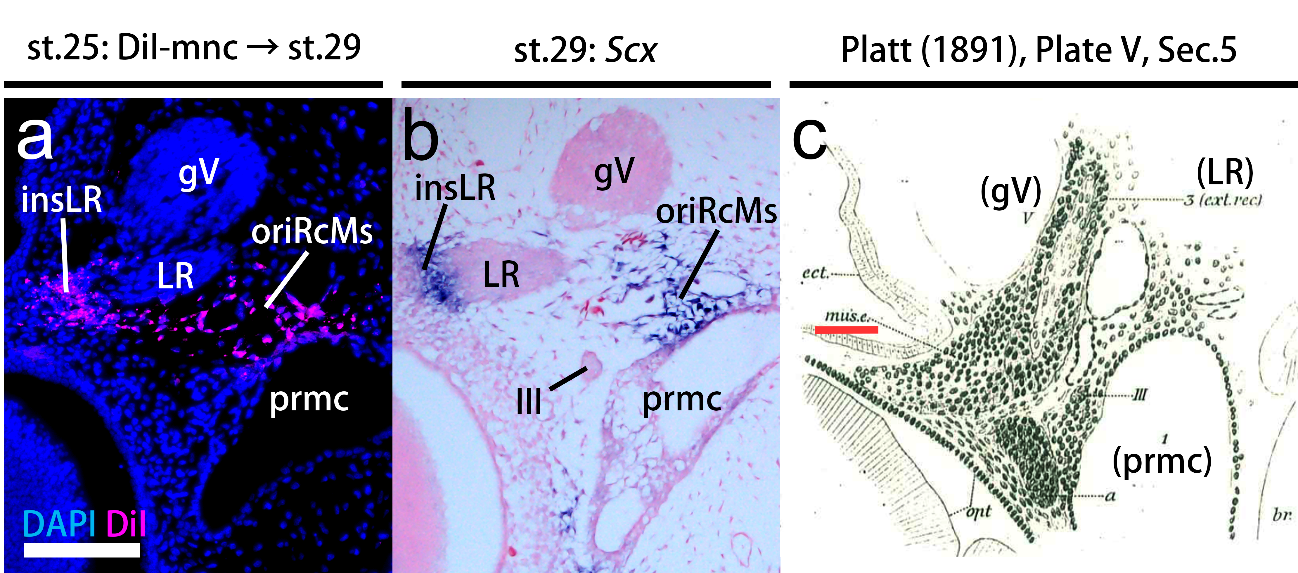
Figure S6. Cell Fates of mnc-derived cells and Platt’s ‘muscle E’. (a)** Medio-lateral distributions of DiI-labelled cells lying continuously from the future points of origins of the rectus muscles to the insertion to the lateral rectus muscle. **(b)** *Scx*-positive cells in a shark embryo at st. 29 show the distribution of the future tendinous cells of EOMs. **(c)** The original plate in ref. [14]. Comparing this plate with (a) and (b), we can see that ‘mus.e.’ (an abbreviation for ‘muscle E’) in this plate (underlined in red by the authors) corresponds to mnc-derived tendon progenitor cells at the insertion of the lateral rectus muscle (insLR). (c) is from ref. [14] with some additional annotations by the authors (presented in brackets).

III, oculomotor nerve; gV, trigeminal ganglia; insLR, tendon progenitor cells at insertion of the lateral rectus muscle; LR, lateral rectus muscle; mnc, mandibular head cavity; oriRcMs, tendon progenitor cells at the origin of rectus muscles; prmc, premandibular head cavity.

Scale bar = 200 µm.

**
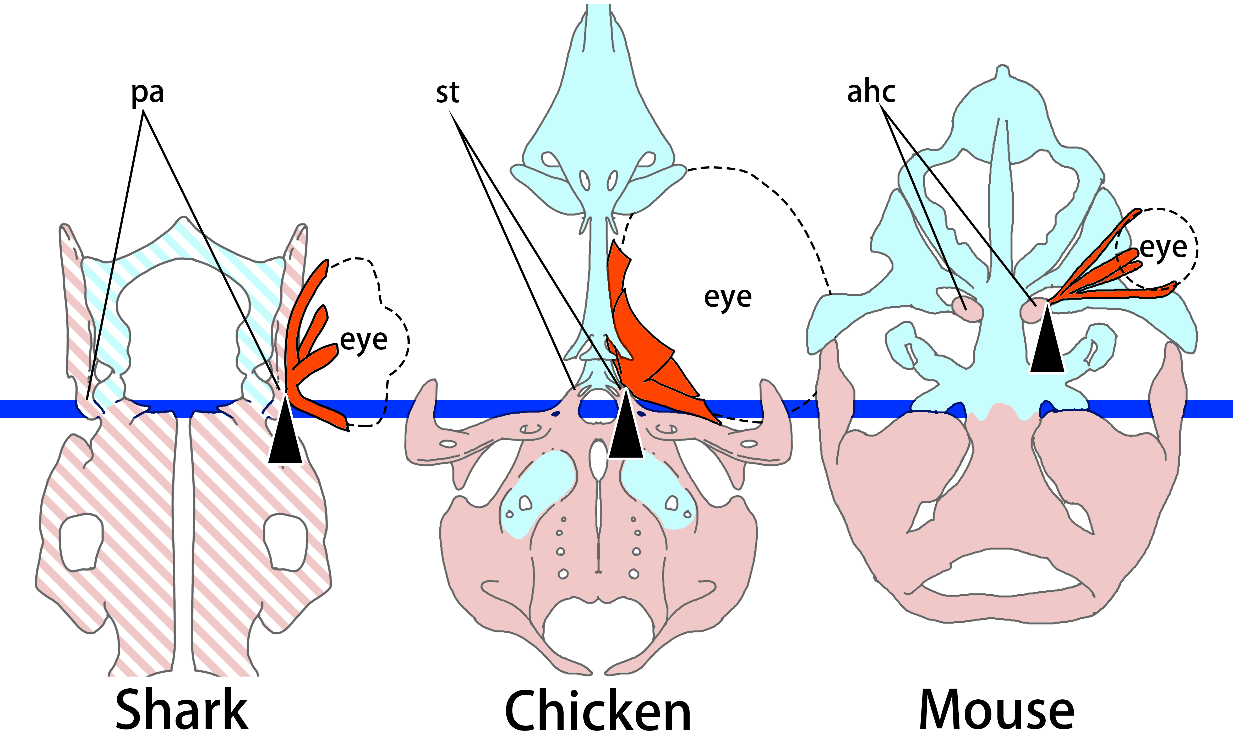
Figure S7. The attachment of rectus components of EOMs on the chondrocranium.** Schematic drawings of the chondrocranium of the shark (ventral view), chicken (dorsal view), and mouse (ventral view) aligned with the position of the hypophysis (blue solid line). The chondrocranium of each animal is colored according to their developmental origin (light blue for the neural crest; pink for the mesoderm). Black arrowheads indicate the proximal attachment sites of the rectus components of EOMs (red). Cell lineages of the chicken and mouse are based on ref. [35, 43] and ref. [56], respectively. Cell lineages of the hatched area in the shark are predicted.

ahc, ala hypochiasmatica; pa, pila antotica; st, supratrabecular cartilage.
